# How and Why Does the Attitude-Behavior Gap Differ Between Product Categories of Sustainable Food? Analysis of Organic Food Purchases Based on Household Panel Data

**DOI:** 10.3389/fpsyg.2021.595636

**Published:** 2021-02-16

**Authors:** Isabel Schäufele, Meike Janssen

**Affiliations:** ^1^Department of Food and Agricultural Marketing, Faculty of Organic Agricultural Sciences, University of Kassel, Witzenhausen, Germany; ^2^Department of Management, Society and Communication, Copenhagen Business School, Frederiksberg, Denmark

**Keywords:** organic food, consumer behavior, attitude-behavior gap, panel data, food-related values, structural equation model, product categories, sustainable food

## Abstract

Organic agriculture promotes the transformation toward sustainability because of positive effects for the environment. The organic label on food products enables consumers to make more sustainable purchasing decisions. Although the global market for organic food has grown rapidly in recent years, only a part of the organic product range benefits from this positive trend. To develop the organic market further, it is important to understand the food-related values and attitudes that drive the purchase of organic food. Previous research on this topic has suffered from two main weaknesses. Firstly, most studies have been based on surveys and rely on stated behavior instead of actual purchase behavior. Secondly, the focus of most extant studies is predominantly on organic food in general or on food products with a relatively high organic market share, such as milk and eggs. To address this knowledge gap, the present study analyzes the value-attitude-behavior relationship by means of structural equation modeling using household purchase panel data from GfK. The paper provides evidence for the existence of an attitude-behavior gap in the organic market, with this gap found to be much stronger in the case of meat, frozen food, cheese, and sweets than for organic purchases in total. Analysis in different product categories reveals that while purchase behavior is driven by the same food-related values, their relative importance differs.

## Introduction

Current food production systems and consumption patterns negatively affect the environment and human health. Climate change, biodiversity loss, and diet-related diseases are severe consequences which call for a shift toward sustainable food systems. A major issue with regard to food production systems are negative environmental effects of agricultural practices, i.e., the use of chemical pesticides and fertilizers. Organic agriculture is one of the most successful certified production standards that promotes the transformation toward sustainability because of positive effects for the environment (Caesar, 2019; Liu and Zheng, 2019; Vermeir et al., 2020) specifically with regards to biodiversity (Reisch et al., 2013). The organic label on food products enables consumers to make more sustainable purchasing decisions (Hsu et al., 2020; Vermeir et al., 2020) driven by growing health and environmental concerns (Hidalgo-Baz et al., 2017).

The global market for organic food has grown rapidly in recent years (Willer et al., 2018), driven in large part by public debates on climate change and biodiversity loss as well as individual concerns about diet-related diseases. Although a significant proportion of consumers are inclined toward organic products and report buying them regularly, only a part of the organic product range benefits from this positive market trend. For example, organic milk, vegetables, and eggs are most successful on the organic market, while organic beverages and organic meat remain niche products (Willer et al., 2018). In order to sustain the growth of the organic market, it is important to understand the factors that drive the purchase of organic food in different product categories. However, the focus of previous studies has been on organic food in general or on food products such as milk and eggs that have a relatively high organic market share, while little is yet known about purchase drivers and barriers in product categories with a low organic market share. When asking consumers directly about their attitudes toward organic food, they often state positive attitudes and purchase intentions (Schäufele and Hamm, 2018).

Numerous studies found a link between positive attitudes and reported purchase behavior (Scalco et al., 2017; Tandon et al., 2020a). Reasons why consumers prefer organic food are its naturalness (Liang and Lim, 2020) and positive effects for health and environment (Dangi et al., 2020; Tandon et al., 2020b). However, most of these studies are based on survey data or laboratory experiments on stated behavior or attitudes and do not analyze actual purchase behavior. Studies that draw conclusions about the drivers of purchase behavior based solely on antecedents of purchase behavior without analyzing actual purchase behavior are potentially biased, moreover, as a result of the well-known attitude-behavior-gap (Aschemann-Witzel and Niebuhr Aagaard, 2014; Hidalgo-Baz et al., 2017) or intention-behavior-gap (Loy et al., 2016). A recent analysis of ElHaffar et al. (2020) reviewed studies on the attitude-intention-behavior gap and revealed how the gap could be reduced and how research could yield more reliable results. One of the most important sources of bias are socially desirable answers, which occur in the absence of any incentives to reveal true attitudes or actual past behaviors (Auger and Devinney, 2007). Using actual purchase data and not just stated behavior is critical, therefore, in order to avoid such bias when seeking to identify the motivating factors of organic food purchases.

Only a few studies to date have made use of actual market data to analyze organic purchase behavior (Janssen, 2018), and of these, only two examined different product categories. Moser (2016) used survey and retail panel data to examine the influence of environmental concerns on purchase behavior in five food categories (chocolate, eggs, meat, milk, and yogurt), albeit without analyzing other drivers. The findings showed that even though environment-related attitudes had an influence on self-reported purchase behavior, no effect on real purchase behavior could be detected in any of the food categories. A study by Van Doorn and Verhoef (2015) used scanner data to analyze organic purchase behavior in 28 product categories, revealing ethical values to be the most important drivers while health-consciousness and quality-consciousness were only found to influence organic purchases in particular categories.

Given the scarcity of knowledge based on real purchase data and conflicting results in the extant literature, further research based on household panel data is required to understand the motivating factors of organic food purchases in different product categories. The continuing expansion of the organic product range in discount stores and supermarkets makes it especially relevant to identify purchase drivers and barriers in order to develop well-targeted marketing strategies to attract new buyers of organic food. Knowledge about how the attitude-behavior gap for organic food differs between product categories and which factors moderate this relation is also important for future research and market actors.

Accordingly, the study strives to answer the following research questions:

- How do food-related values affect consumers' organic food purchases, i.e., which food-related values serve as drivers/barriers of organic food purchases?
- What is the mediating role of attitudes toward organic food within the value-behavior relation?

To address the research questions, we estimated a structural equation model capturing expenditures for organic food across all food categories. Since the existing body of literature suggests that certain food values exert different effects on different product categories, we also ran separate models for four product categories with persistent low shares in the organic market: cheese, meat, frozen food, and sweets. The study thus includes a range of different product categories, e.g., in terms of hedonic consumption and types of food.

## Theoretical Framework: Values, Attitudes, and Behavior

The concept of motivation is closely interlinked with the concept of values and attitudes (Solomon et al., 2006). Values, understood here as comprising a person's beliefs about what constitute desirable states and behaviors beyond any particular circumstances, have been shown to relate closely to attitudes that motivate purchase decisions (Vinson et al., 1977; Schwartz, 1992; Rohan, 2000). While many theories have been developed regarding values and/or attitudes and their influence on behavior (Schwartz, 1994; Stern et al., 1999; Zepeda and Deal, 2009), little is yet known about the value-attitude-behavior chain in the case of organic food purchase behavior.

According to Vinson et al. (1977), three different levels of values can be distinguished: global values, domain-specific values, and evaluative beliefs. Global values comprise a person's most “centrally held and enduring beliefs” and thus “form the central core of an individual's value system” (Vinson et al., 1977), existing thus at an abstract level and influencing actions and evaluations beyond any specific situations. Domain-specific values are less closely held and less generalizable values acquired through a person's experiences of “specific situations or domains of activity” (Vinson et al., 1977). Evaluative beliefs, the third and most numerous category in this model of values, refer to a person's least centrally held and most specific beliefs, sometimes considered equivalent to the concept of attitudes (Honkanen et al., 2006).

In the context of food purchases, several studies have provided evidence of the important role of values as predictors of behavior (e.g., Connors et al., 2001; Lusk and Briggeman, 2009; Lusk, 2011; Hauser et al., 2013). Investigating the importance that consumers place on different characteristics of food and food production (e.g., healthiness, taste, price, and environment-friendly production), these studies refer to such values in various terms, including “domain-specific values” (Honkanen et al., 2006) “food values” (Lusk, 2011), “food-related values” (Hauser et al., 2013) “motives related to food choice” (Steptoe et al., 1995), and “food choice motives” (Eertmans et al., 2005).

The consumer behavior literature further postulates that attitudes are the central concept by which to explain behavior. Attitudes here refer to a person's long-term evaluations of objects to satisfy particular motives. Consumers' attitudes to products are developed through beliefs (cognition) and feelings (affect) about a product. Attitudes as such may consequently translate into actual behavior, although a direct attitude-behavior relation has been shown to hold true only under certain conditions (Solomon, 2015). The predictive power of simple attitude-behavior models has been much improved upon through the incorporation of several additional constructs in Ajzen's Theory of Planned Behavior (Ajzen, 1985), now one of the most prominent theories in social psychology. In this model, values act as background variables that influence behavior indirectly through their effect on attitudes, hence attitudes are seen as fully mediating the value-behavior relation. Many studies on organic food consumption have applied the Theory of Planned Behavior, though most have analyzed only purchase intention for generic organic food rather than real purchase behavior for specific organic food categories (Scalco et al., 2017).

Hauser et al. (2013) have further enhanced Ajzen's approach, demonstrating that values exert both indirect and direct effects on food purchase behavior. In this view, the purchase of food is not completely cognitively controlled but rather executed habitually; and therefore attitudes do not fully mediate the relation between values and behavior; values can have significant direct effects on behavior.

## Literature Review: Food-related Values and Organic Food Consumption

A large body of literature has examined the links between consumers' values and organic food consumption to understand why consumers choose or not choose organic food, though again it should be noted that the great majority of studies have analyzed survey data rather than actual purchase data. This section gives an overview of the state of the art on food-related values and organic food consumption. Table 1 summarizes the results of the literature review according to the direction and strength of effect of different food-related values on organic food consumption.

**Table 1:** Literature review: food-related values and organic food choice.

**Food-related values**	**Direction and strength of the effect on organic food consumption according to the number of studies that support the relation**
Healthiness and naturalness	++
Environmental protection	++
Animal welfare	++
Local and domestic food	+
Convenience orientation	-
Quality and enjoyment	°
Price consciousness	- -

^+^*Positive relation*, ^−^*Negative relation*, °*Contradictory findings*.

^++/−−^*Strong evidence*, ^+^/^−^*Weak evidence*.

Most studies have found that organic food consumption is motivated in large part by consumers' health values, with organic food perceived as healthier and more natural than conventional food on account of being free from chemical residues and artificial additives (Janssen, 2018; Rana and Paul, 2020). Ethical values have also been found to play a key role as drivers of organic food consumption, in particular, the values of environmental protection (Janssen, 2018; Rana and Paul, 2020) and animal welfare (Padilla Bravo et al., 2013; Van Doorn and Verhoef, 2015). Several studies have also shown a positive link between values for local food and organic food consumption (Padilla Bravo et al., 2013; Hempel and Hamm, 2016) and a preference for fresh food, i.e., a negative effect of convenience values on the consumption of organic food (Hauser et al., 2013; Janssen, 2018). Numerous studies have further shown that organic consumers are less price-conscious than most other consumers (e.g., Janssen, 2018).

Regarding the effect of quality and enjoyment orientation on organic food consumption, however, previous studies have produced contradictory results (Nadricka et al., 2020). Some research has shown that consumers of organic food prefer high quality and exclusive food (Padilla Bravo et al., 2013; Rana and Paul, 2020). Other studies have not been able to find any effect of quality and enjoyment orientation on the purchase of organic food (Van Doorn and Verhoef, 2015), or have even found that consumers with high quality and enjoyment orientation buy less organic food (Hauser et al., 2013). A possible explanation for these conflicting results regarding consumers' quality orientation may be related to product-specific differences in preferences for organic products (Nadricka et al., 2020). For example, a study by Van Doorn and Verhoef (2011) has shown that consumers associate organic production with lower quality when it comes to products that promise immediate pleasurable experience, such as cheese, chips, salty biscuits, chocolate, cookies, pastries, and candy, etc. The authors argue that the “healthiness” of organic production actually reduces the pleasure experienced in the consumption of such “vice” or pleasure foods. Similar results were obtained by Rousseau (2015) in a study on organic chocolate. A study on organic cheese also found that consumers' preference for organic production was relatively low compared to the decisive role played by taste and place of origin in their purchase of cheese (Bernabéu et al., 2010).

Another finding relevant to understanding the attitude-behavior gap in relation to the purchase and consumption of specific organic food products was made in a study about biscuits by McIntyre and Schwanke (2010). This study concluded that the added value of organic production was unable to compensate consumers for what they perceived as the inherent “unhealthiness” of biscuits. Like other health attributes, organic production presented no extra value to consumers as compared to the sensory properties of this product, which were largely perceived as decisive for this “treat” product. Organic labeling alone thus seems insufficient to compete with enjoyment as the main reason for buying biscuits. Indeed, the organic attribute was even perceived as undesirable in the case of this product, in strong contrast to the effect of this quality on the consumption of organic raw products such as vegetables and meat, where the benefits of organic production are more evident to consumers (McIntyre and Schwanke, 2010). A study by Arvola et al. (2008) comparing consumers' preferences for organic apples and organic pizza similarly concluded that products associated with high levels of processing are found incongruent with consumers' ideas of organic production.

The studies cited above were published several years ago. The availability and variety of organic food has generally increased during the past 10 years in many countries. However, also recent research points to interesting differences between consumers' taste perceptions of healthy and unhealthy organic food products. Nadricka et al. (2020) found that healthy food is perceived to be tastier when it has an organic label (compared to the same food without an organic label). For unhealthy food, however, this effect was not observed. The authors were able to show that the effect of organic labels on taste evaluations is explained by perceived healthiness of the product category. It is thus interesting to re-visit the relationship between quality and enjoyment orientation and actual organic food purchases with more recent data and across different food categories.

## Materials and Methods

### Dataset and Definition of Variables

The present study is based on two consumer panels of the GfK market research institute: ConsumerScan (which includes the purchase of packaged food) and ConsumerScan FreshFood (which includes unpackaged food). The dataset consisted of 8,400 households in Germany who participated in both panels throughout 2016 (The final sample comprised 8,065 households due to missing values in survey questions). The data covered total organic and conventional food purchases aggregated at household level, including specific information on the purchase of organic and conventional cheese, meat, frozen food, and sweets.

The households continuously recorded their food purchases by scanning the European Article Number (EAN) code, which provides specific information about products, including whether they are organic. The participants additionally specified the prices and quantities of each product they bought. On the basis of this information, the variable “organic budget share” (OBS) was computed as a measure for households' organic food purchases. A household's OBS is thus calculated (in euros) as the ratio of their expenditure on organic food to their total expenditure on food over the 12 months of 2016. The variable thus takes on values within an interval bounded from 0 for households that buy no organic food to 1 for households that buy exclusively organic food. In addition to overall (organic) food expenditures, we calculated the OBS for specific product categories, i.e., the ratio of expenditure (in euros) on organic cheese, meat, frozen food, and sweets to total expenditures within the respective product categories.

The data also included socio-demographic information. “Income” was calculated as the weighted monthly net income per household member, and this variable comprised five classes. “Education” was defined by the highest school qualification of the diary keeper (the person in the household responsible for the purchase diary), and a dummy variable was created with the value 1 for holding at least a university entrance diploma. “Age” referred to the age of the person responsible for the households' food purchases.

In addition to actual food purchase data and socio-demographic information, the dataset included 130 survey items on food-related values and attitudes toward organic food from an annual written and self-administered questionnaire. The level of participants' agreement with the survey items was indicated through a five-point rating scale from (1) “I do not agree at all” to (5) “I totally agree.” One item on the topic of animal welfare used a six-point rating scale from (1) “That's not relevant to me” to (6) “I have done so in the past/I already do that today.”

Three items were used to measure consumer attitudes to organic food (similar to those used in Janssen, 2018): “When buying food, I prefer organic food”; “I would like to see a larger assortment of organic food in grocery stores”; and “I am willing to pay higher prices for organic food.” In the next step, we selected potential indicators for the value constructs we hypothesized would be linked to organic food purchases: healthiness and naturalness; environmental protection; animal welfare; local and domestic food; convenience orientation; quality and enjoyment; and price consciousness (see Table 1). For two of the seven constructs, the GfK survey only contained one statement per item: price-consciousness (“When buying food, I care more for prices than for brands”) and animal welfare (“I prefer to buy/eat meat from animal-friendly production systems”). These constructs were included in the structural model as single-item constructs. The remaining five constructs were each assigned three or more potential indicators. With an exploratory factor analysis (Eigenvalue > 1, Varimax rotation), we analyzed whether the constructs could be considered as distinct dimensions (= uncorrelated factors) and whether the indicators proved to be assigned to the constructs (= factors) as expected. The analysis resulted in a seven-factor solution instead of the expected five factors. The seven factors partly overlapped in terms of content. In the next step, we reduced the number of items and omitted those that formed factors difficult to interpret. The second EFA resulted in a six-factor solution. Two items we had expected to allocate to the factor “environmental protection” were found to constitute separate factors by themselves, and two of the 28 items (“I only buy fresh products instead of e.g., canned or frozen food” and “For the preparation of food I prefer fresh ingredients”) had factor loadings smaller than 0.5. These four items were therefore excluded and a third factor analysis was run. The scale-reliability test showed that one item significantly reduced the reliability of the respective factor. This item was therefore removed and a fourth factor analysis was conducted. In the final solution, the factor structure and distribution of items was in accordance with prior expectations. The five factors explained 55.5 % of the total variance. This solution was used in the subsequent confirmatory factor analysis (CFA).

### Structural Equation Modeling

**Figure 1:**
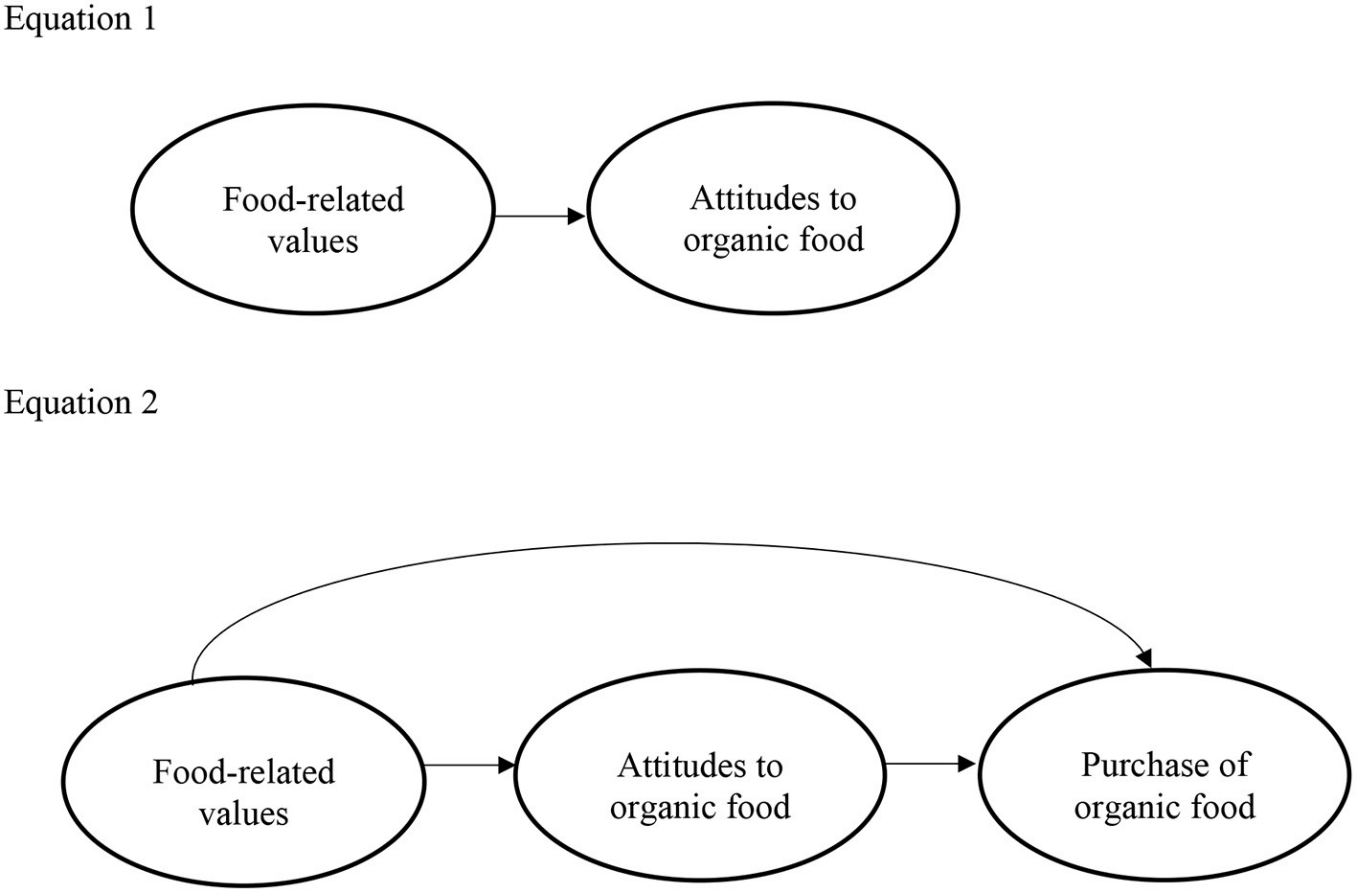
Structural equation model.

The data were analyzed with the lavaan R software package for Structural Equation Modeling (SEM) (Rosseel, 2012). A comprehensive and flexible multivariate data analysis method that estimates relations between variables, SEM encompasses a structural model and a measurement model. The measurement model defines latent constructs, such as attitudes and other psychological constructs that are not directly observable, by several observable variables through CFA (Gana and Broc, 2019; Hair et al., 2019). The structural model applies multiple regression analysis methods and estimates a sequence of distinct but interdependent multiple regression equations simultaneously. In this model, variables can have a reciprocal role, i.e., a dependent variable in one equation can become an independent variable in other parts of the SEM (Gunzler et al., 2013). In the present study, “attitudes toward organic food” have the role of such a two-sided variable. Equation 1 investigates the effect of food-related values on attitudes, while Equation 2 specifies attitudes as the “mediator” variable between food-related values and behavior (see Figure 1). Both equations further controlled for the socio-demographic variables of income, education, and age.

From a theoretical perspective, a mediator variable can serve to explain why a relationship between two constructs exists (Baron and Kenny, 1986); it can be regarded as an “intervening or facilitating variable” (Hair et al., 2019, p. 745). To demonstrate mediation in our case, we needed to observe strong relations between food-related values (= the exogenous variables) and attitudes (= the mediating variable), and between attitudes and behavior (= the endogenous variable). We did not hypothesize that attitudes would fully mediate the effect of food-related values on behavior, but we expected a partial mediation so that food-related values would also have direct effects on behavior.

SEM is a covariance analysis structure technique that explains covariation among variables. The weighted least squares mean and variance adjusted (WLSMV) estimation method was chosen because the normality assumption was violated and the model included ordinal variables. The WLSMV estimation method is a robust version of Weighted Least Squares estimation methods (Gana and Broc, 2019) and substitutes the full weight matrix by a weight matrix that contains only diagonal elements, meaning only asymptotic variances and polychoric correlations are included (Moshagen and Musch, 2014).

## Results

### Description of the Sample

The socio-demographic characteristics of the sample and the German population are presented in Table 2. Direct comparison is difficult because the federal statistical office applies different age and income categories. Moreover, the education categories of the household survey involve a combination of school-leaving and vocational qualifications, whereas the German federal office provides two separate statistics on these types of educational qualification.

**Table 2:** Socio-demographic characteristics of the sample and the German population.

**Socio-demographics** **(*N* = 8,400)**	**Sample%**	**Population%**	
	**Age of the head of household**	**Age of German residents older than 18 years^a^**	
Up to 29 years	1.9	17.0	
30–39 years	10.1	14.2	
40–49 years	17.2	19.9	
50–59 years	24.8	50 up to under 75 years	
60–69 years	23.3	37.8	
70 years and older	22.6	75 years and older 11.2	
	**Formal education of the diary keeper (including vocational school and university)**	**School-leaving qualification of German residents^b^**	**Vocational qualification of German residents^c^**
Secondary general school	22.5	29.6	-
Intermediate secondary school	32.9	29.9	-
Qualified dual vocational training programme	-	-	47.5
Special upper secondary school (vocational school)	8.0	-	8.8
University entrance diploma	14.1	32.5	-
University	22.5	-	18.0
Others	-	8	25.7
	**Household net income**	**Net income of private households in Germany^d^**	
up to 749 Euro	3.5	Under 1,500 26%	
750–1,249 Euro	12.9		
1,250–1,749 Euro	16.2	1,500–3,200 43%	
1,750–2,249 Euro	18.8		
2,250–2,749 Euro	15.6		
2,750–3,249 Euro	12.8		
3,250–3,749 Euro	7.7	Over 3,200 31%	
3,750–4,999 Euro	9.2		
5,000 Euro and more	3.3		

a *German Federal Statistical Office (2020), table 12111–0004*.

b *German Federal Statistical Office (2019), p. 88*.

c *German Federal Statistical Office (2019), p. 90*.

d *German Federal Statistical Office (2020), table 12111-0004*.

With regard to age, young households were underrepresented in the sample, in particular the youngest age group (2% in the sample vs. 17% in the total population). In about a third of the households, the diary keeper (person responsible for the purchase diary) held a university-entrance diploma or a university degree, which is quite similar to the distribution of the highest school-leaving qualification of the German population. The data further suggests that high-income households were underrepresented in the sample.

Table 3 presents the distribution of the organic budget share (OBS) for food overall and for specific food categories among the households. With regard to food overall, almost all households (96.5%) bought at least some organic food in 2016 (i.e., only 3.5% did not buy a single organic food item). However, a large proportion of the households (40.8%) had an overall OBS below 1%, thus it is assumed that these households' purchase of organic items may have happened unintentionally. Some 37.1% of the households rarely purchased organic food (OBS 1% to <5%). While 14.7% of households once in a while purchased organic food, these households still spent the great majority of their food budget on conventional food (OBS of >5–20%). Only 4.0% of the panel households spent a significant part of their food budget on organic food (OBS of >20%). Focusing on individual food categories, it is striking that the great majority of the households (between 73 and 82% depending on the product category) did not buy any organic cheese, meat, frozen food, or sweets in the 12-month period. Correspondingly, the proportion of households falling in the categories of occasional and frequent buyers of organic food was much lower in individual food categories compared to the distribution of the overall organic budget share. Interestingly, however, the proportion of households who spent a significant amount of their food category budget (OBS of >20%) on organic cheese, meat, and frozen food did not deviate substantially between the food categories and overall expenditures on food, with 3.9, 6.2, and 2.3% of households classifying as regular buyers of organic cheese, meat, and frozen food. However, regular buyers of organic sweets were very rare, amounting to only 1.5% of households.

**Table 3:** Annual organic budget share (OBS) within each food category.

	**Share of respondents in %**				
**Annual organic budget share**	**Food overall**	**Cheese**	**Meat**	**Frozen food**	**Sweets**
0%	3.5	73.1	81.8	73.4	75.7
>0– <1%	40.8	2.3	0.7	4.2	7.2
1– <5%	37.1	12.7	5.1	13.2	11.0
5– <20%	14.7	8.0	6.2	6.9	4.6
≥20%	4.0	3.9	6.2	2.3	1.5
Total	100.0 (*N* = 8,400)	100.0 (*N* = 8,340)	100.0 (*N* = 8,140)	100.0 (*N* = 8,253)	100.0 (*N* = 8,379)

Table 4 demonstrates the relationship between the OBS and overall expenditures (in euros) within the specific food categories (organic and non-organic products together). This shows that the higher a household's OBS, the more they spent on average on cheese, i.e., both organic and non-organic cheese. The expenditure share for cheese in relation to overall food expenditures increased with the OBS as well, i.e., households with a high OBS spent a relatively high share of their overall food expenditure on cheese.

**Table 4:** Expenditures for food categories by annual organic budget shares.

	**Cheese**		**Meat**		**Frozen food**		**Sweets**	
**Annual organic budget share**	**Average expenditures on cheese (in Euros)**	Average expenditure share for cheese%^*^	**Average expenditures on meat (in Euros)**	Average expenditure share %^*^	**Average expenditures on frozen food (in Euros)**	Average expenditure share %^*^	**Average expenditures on sweets (in Euros)**	Average expenditure share %^*^
0%	64.35^a^	3.9^a^^,^^b^	112.93^a^	6.0^a^^,^^b^	88.08^a^	5.0^a^	130.18^a^	7.7^a^
>0– <1%	92.97^b^	3.7^a^	165.53^b^	6.1^a^	121.43^b^	4.6^a^	179.53^b^	7.1^a^
1– <5%	108.92^c^	4.2^b^	150.78^c^	5.4^b^	110.38^c^	4.1^b^	170.54^c^	6.6^b^
5– <20%	121.85^d^	4.6^c^	137.51^c^	4.6^c^	93.86^a^	3.3^c^	151.38^d^	5.7^c^
≥20%	162.24^e^	5.3^d^	157.18^b^^,^^c^	4.5^c^	84.11^a^	2.7^d^	147.54^d^^,^^a^	4.7^d^
	*N* = 8,400							

* *In relation to overall food expenditures*.

a,b,c,d,e *Within each column, average expenditures, and average expenditure shares with different letters are significantly different from each other (p < 0.05)*.

With meat, frozen food, and sweets, the exact opposite relation was found. In these product categories, households with a higher organic budget shares spent significantly lower shares of their food expenditures on meat, frozen food, and sweets than households with low OBS. For instance, households who purchased no organic food in 2016 spent 6.0, 5.0, and 7.7% of their food expenditures on meat, frozen food, and sweets, respectively, while for households with a high OBS these shares amounted to 4.5, 2.7, and 4.7%.

Table 5 displays the answer distribution of the three attitude items on organic food. More than 20% of households partly or fully agreed with the following statements: “When buying food, I prefer organic food” (23%); “I am willing to pay higher prices for organic food” (24%); and “I would like to see a larger assortment of organic food in grocery stores” (28%). The positive stated attitudes of around one-fourth of the sample, when compared to the fact that only 4% of the households spent more than 20% of their food budget on organic food (Table 3), clearly shows an attitude-behavior gap applies in a considerable share of households.

**Table 5:** Attitude statements on organic food^a^.

	**I fully disagree**	**I rather disagree**	**Neither nor**	**I rather agree**	**I fully agree**	**Total**
When buying food, I prefer organic food	27.8	26.6	22.8	17.0	5.7	100.0
I am willing to pay higher prices for organic food	25.2	22.1	28.5	17.4	6.8	100.0
I would like to see a larger assortment of organic food in grocery stores	27.0	21.3	23.5	21.7	6.5	100.0
						*N* = 8,137

a *The three statements form the construct “Attitudes toward organic food” (see Tables 6, 7)*.

### Evaluation of the Measurement Model

The measurement model was evaluated by assessing discriminant validity and convergent validity, which is the common procedure in SEM (Hair et al., 2019). Discriminant validity was assessed through the Fornell-Larcker criterion (Averages variance extracted (AVE) > squared correlation with any other construct) to make sure that the factors identified in the exploratory factor analysis were truly distinct from one another. This was the case with all factors in our model.

Convergent validity specifies the extent to which the items within the same construct share a high proportion of variance in common. Convergent validity was assessed by factor loadings, construct reliability, and average variance extracted (AVE):

- The factor loadings of three items were below 0.5 and these items were therefore removed: “I pay attention to what I eat and drink because I need to take care of my health” (construct healthiness and naturalness); “The government and the industry, not ordinary citizens, should take care of protecting the environment” (construct environmental protection); “Frozen food is just as good as fresh food” (construct convenience orientation). Finally, the factor loadings of all items were above 0.5 and thus, satisfactory (see Table 6).
- Construct reliability—a reliability measure commonly used in SEM instead of Cronbach's alpha—was computed from the squared sum of factor loadings for each construct and the sum of the error variance terms for a construct (Hair et al., 2019). High construct reliability of >0.7 indicates that internal consistency exists. In our case, all values were higher than 0.8 (see Table 7) and thus, good. We also included Cronbach's alpha in Table 7, since some readers might be more familiar with this reliability measure.
- Finally, the AVE was examined. In SEM, the AVE is calculated as the mean variance extracted for the items loading on a construct; i.e., AVE equals the sum of all squared factor loadings divided by the number of items in the construct. This indicator should be higher than 0.5. Three constructs did not achieve this threshold (Hair et al., 2019). However, the measurement model was accepted because of good levels of construct reliability, factor loadings, and discriminant validity.

**Table 6:** Confirmatory factor analysis.

**Constructs**	**Indicators**	**Standardized factor loading**
Healthiness and naturalness	I dislike products containing preservatives	0.78
	When shopping for food, I am careful to choose products without any additives	0.82
	I dislike products containing flavor enhancers (e.g., glutamate)	0.62
	I obtain information about which food is environmentally polluted and stop buying it	0.67
	In my diet, I avoid everything that is bad for my health.	0.52
Convenience orientation	Ready-made refrigerated meals are as good as self-made meals	0.74
	Nowadays, canned food tastes as good as fresh food to me	0.75
	I can hardly image cooking without convenience products (like instant gravy, frozen food, canned food).	0.65
Local and domestic food	When I have the choice, I definitely buy food from Germany	0.82
	For me, food from Germany has the best quality	0.65
	When I have the choice, I prefer local food	0.84
	I am willing to pay more for local products	0.76
Environmental protection	Nowadays, too much fuss is made about the environment	0.76
	What is currently done to protect the environment is absolutely sufficient	0.65
	In my household, I can do little to protect the environment	0.50
	I care little about the environmental impact of products	0.69
Quality and enjoyment	I like treating myself to fine food	0.59
	I treat myself to delicacies once in a while.	0.57
	I like cooking extravagant dishes	0.63
	I demand high standards when it comes to food and drinks	0.71
Attitudes toward organic food	When buying food, I prefer organic food	0.95
	I would like to see a larger assortment of organic food in grocery stores	0.94
	I am willing to pay higher prices for organic food	0.93

**Table 7:** Construct validity and reliability.

	**Average variance extracted**	**Construct reliability**	**Cronbach's alpha**
Healthiness and naturalness	0.48	0.88	0.80
Convenience orientation	0.51	0.85	0.73
Local and domestic food	0.59	0.90	0.83
Environmental protection	0.43	0.84	0.72
Quality and enjoyment	0.39	0.88	0.70
Attitudes toward organic food	0.88	0.95	0.94

### Structural Model

The goodness-of-fit indices, i.e., the “Standardized Root Mean Square Residual (SRMR),” the “Root-Mean-Square-Error of Approximation (RMSEA),” the “Comparative Fit Index (CFI),” and the “Tucker-Lewis Index (TLI),” indicate that all models fit the data well (Gana and Broc, 2019) (see Table 8).

**Table 8:** Goodness-of-fit indices of the structural model.

	**Food overall**	**Cheese**	**Meat**	**Frozen food**	**Sweets**
**indicator**					
RMSEA	0.063	0.084	0.085	0.084	0.084
p-value (RMSEA < 0.05)	0.000	0.000	0.000	0.000	0.000
SRMR	0.056	0.045	0.045	0.045	0.045
TLI	0.931	0.963	0.959	0.963	0.963
CFI	0.909	0.954	0.949	0.954	0.954

The model for organic food purchases across all food categories explained 28.8% of the variance of the construct “organic purchases.” For cheese (14.0%), sweets (10.8%), meat (9.6%), and frozen food (8.7%), the explained variance in organic food purchases was relatively low (see **Table 10**). The explained variance in the mediator construct “attitudes toward organic food” was comparably high at 52.6%. Thus, the independent variables better explained attitudes than real purchase behavior. Among the independent variables, socio-demographics had a low explanatory power compared to food values for both dependent constructs. Education and income had a positive effect on organic purchases, while age exerted a negative effect (see Table 9).

**Table 9:** Effects on organic purchases and attitudes toward organic food^a^.

	**Organic purchases (food overall)*^b^***						**Attitudes toward organic food**	
	**Direct effects**		**Indirect effects**		**Total effects**		**Direct effects=total effects**	
Healthiness and naturalness	0.03	^*^	0.15	^***^	0.18	^***^	0.33	^***^
Convenience orientation	−0.10	^***^	0.00	n.s.	−0.09	^***^	0.00	n.s.
Local and domestic food	−0.07	^***^	0.08	^***^	0.01	n.s.	0.17	^***^
Environmental protection	0.07	^***^	0.08	^***^	0.15	^***^	0.18	^***^
Quality and enjoyment	−0.05	^***^	0.01	n.s.	−0.05	^*^	0.02	n.s.
Price consciousness (single indicator)	−0.07	^***^	−0.08	^***^	−0.15	^***^	−0.18	^***^
Animal welfare (single indicator)	−0.02	^*^	0.18	^***^	0.17	^***^	0.40	^***^
Attitudes toward organic food	0.46	^***^						
Age	−0.04	^***^	−0.02	^***^	−0.07	^***^	−0.05	^***^
Education	0.04	^***^	0.05	^***^	0.09	^***^	0.10	^***^
Income	0.05	^***^	0.02	^***^	0.07	^***^	0.05	^***^
Variance extracted			28.8%				52.6%	
*N*	8,065							

*** *Significant at p < 0.001*.

** Significant at p < 0.01.

* *Significant at p < 0.05*.

*n.s., not significant*.

a *Results of structural equation modeling on the values-attitude-behavior relation (see Figure 1)*.

b *Organic purchases (food overall) refers to the ratio of expenditures for organic food (in €) to the total expenditures for food (in €) during the 1 year period 2016*.

Here, the total effects of total organic purchases are presented first, after which the differences among the specific food categories are explained, followed by a comparison of the direct and indirect effects in the different models. The constructs “healthiness and naturalness,” “animal welfare,” “environmental protection,” and “price-consciousness” all had a significant influence on total organic purchases, i.e., households who cared more for healthy and natural food, animal welfare, and environmental protection had a higher organic budget share. Higher price-consciousness, however, was associated with a lower organic budget share. The constructs “quality and enjoyment” and “convenience orientation” showed a weak negative influence, while no significant effect at all was found for “local and domestic food.” Comparison of the determinants of attitudes and purchase behavior revealed interesting differences: for example, the construct “animal welfare” had by far the strongest effect on “attitudes toward organic food,” while “healthiness and naturalness” and “animal welfare” were of equal importance for determining the actual purchase of organic food (see Table 9).

Interestingly, the total effects of the independent constructs on organic food purchases differed across food categories. The purchase of organic cheese was mainly determined by consumers' price consciousness (negative effect) and concern for animal welfare (positive effect). The constructs “healthiness and naturalness” and “environmental protection” were also significant, though their effect strength was slightly smaller. For organic meat, animal welfare was by far the most important determinant, while all other food-related values only had a weak impact on the purchase of organic meat. For organic frozen food, “healthiness and naturalness” was most important, followed by “convenience orientation” (negative effect), and “price consciousness.” With regard to the purchase of organic sweets, “healthiness and naturalness” had by far the greatest influence on purchase behavior, followed by “environmental protection,” and “price consciousness” (see Table 10).

**Table 10:** Total effects on organic purchases for different food categories^a^.

	**Food overall**		**Cheese**		**Meat**		**Frozen food**		**Sweets**	
Healthiness and naturalness	0.18	^***^	0.11	^***^	0.05	^**^	0.12	^***^	0.17	^***^
Convenience orientation	−0.09	^***^	−0.07	^***^	−0.04	^*^	−0.09	^***^	−0.03	^*^
Local and domestic food	0.01	n.s.	0.02	n.s.	0.01	n.s.	−0.02	n.s.	−0.02	n.s.
Environmental protection	0.15	^***^	0.10	^***^	0.06	^***^	0.06	^***^	0.12	^***^
Quality and enjoyment	−0.05	^*^	−0.03	^*^	−0.01	n.s.	−0.02	n.s.	−0.02	n.s.
Price consciousness (single indicator)	−0.15	^***^	−0.13	^***^	−0.09	^***^	−0.09	^***^	−0.10	^***^
Animal welfare (single indicator)	0.17	^***^	0.12	^***^	0.20	^***^	0.05	^***^	0.04	^***^
Attitudes toward organic food	0.46	^***^	0.29	^***^	0.21	^***^	0.24	^***^	0.25	^***^
Age	−0.07	^***^	−0.03	^*^	−0.02	n.s.	−0.03	^*^	−0.07	^***^
Education	0.09	^***^	0.07	^***^	0.05	^***^	0.05	^***^	0.06	^***^
Income	0.07	^***^	0.06	^***^	0.03	^**^	0.05	^***^	0.02	n.s.
Variance extracted	28.8%		14.0%		9.6%		8.7%		10.8%	
*N*	8.065		8.008		7.811		7.924		8.046	

*** *Significant at p < 0.001*.

** *Significant at p < 0.01*.

* *Significant at p < 0.05*.

*n.s., not significant*.

a *Results of structural equation modeling on the values-attitude-behavior relation (see Figure 1)*.

Attitudes toward organic food acted as a mediator between food values and purchase behavior. However, significant direct effects reveal that this relation is not fully mediated through attitudes and that most variables also influence behavior directly. The model for total organic purchases showed significant direct effects for all independent variables, while some variables of the food category-specific models influenced behavior only indirectly through attitudes (direct and indirect effects of the different food categories can be found in the Supplementary Material).

For the construct “convenience orientation,” no mediation effect of attitudes was found in either of the food categories. This is linked to the fact that consumers' convenience orientation did not influence their attitudes toward organic food. However, this construct did exert a negative direct and total effect on behavior.

The construct “quality and enjoyment” had a significant (but small) negative effect on organic purchases overall and on the purchases of organic cheese, while no significant total effect on purchases of organic meat, frozen food, and sweets were recorded. Like “convenience orientation,” this construct had no significant effect on attitudes toward organic food.

In the case of the construct “local and domestic food,” quite the opposite was true, with attitudes positively influenced while purchase behavior was not affected in total. This non-significant total effect was due to positive indirect effects and negative direct effects (no significant direct effects for meat).

## Discussion and Conclusions

The present study has shown that food-related values are good predictors of attitudes toward organic food; attitudes in turn play a major role in explaining organic food consumption, consistent with the findings of a recent meta-analysis that attitudes exert the strongest summary effect on behavior within the framework of the Theory of Planned Behavior (Scalco et al., 2017). This leads to the conclusion that attitudes toward organic food are important antecedents of organic food purchases and very good predictors of consequent behavior.

However, our data also revealed that approximately one in four consumers in our sample of more than eight thousand consumers held very positive attitudes toward organic food, but only a small proportion of consumers (4% of all households) directly translated these attitudes into purchase behavior. This study thus provides further evidence of the attitude-behavior gap (Yamoah and Acquaye, 2019; ElHaffar et al., 2020). The following discussion provides possible explanations for the attitude-behavior gap in the different product categories, based on our findings of how food-related values serve as drivers and barriers to organic food purchases (first research question), and how attitudes mediate the value-behavior relation (second research question).

### Drivers and Barriers of Organic Food Purchases and the Attitude Behavior Gap

Interestingly, our household panel data showed that the attitude-behavior gap is much stronger for meat, frozen food, cheese, and sweets than for total organic purchases. The gap between attitudes and behavior can probably partly be attributed to the relatively high price premiums and low availability of organic food in these specific product categories in conventional supermarkets (Vermeir and Verbeke, 2006; Aschemann-Witzel and Niebuhr Aagaard, 2014; Yamoah and Acquaye, 2019). Price-conscious consumers buy less organic food because of the organic premium price (Aschemann-Witzel and Zielke, 2017), and our study shows that for cheese and meat, high price premiums in particular deter consumers from purchasing organic alternatives. High convenience orientation is also connected to low organic budget shares according to our data. Interestingly, convenience orientation had no significant effects on attitudes toward organic food in either of the food categories but it was significantly linked to purchase behavior. This result suggests the attitude-behavior gap can (partly) be explained by the fact that convenience-oriented consumers may not be willing to invest high search costs, and therefore, the low availability of organic food in conventional supermarkets may play a crucial role as a purchase barrier (Gottschalk and Leistner, 2013). However, this proposition requires further investigation since we did not analyze where the households purchase organic food (e.g., in conventional supermarkets or specialized organic food stores).

Confirming the results of previous studies (Dangi et al., 2020; Tandon et al., 2020b), the present study also suggests that organic products are purchased in particular by health-conscious and environmentally conscious consumers. This rather small target group might represent a challenge for making organic food attractive to a broader audience (Van Doorn and Verhoef, 2015), specifically because in product categories like frozen food, sweets, and fresh meat, organic food is not widely available in conventional grocery stores (Rana and Paul, 2017). Interestingly, the present study shows that the motives driving consumers to buy organic food are very similar across the different food categories analyzed, although the relative importance of the drivers differs. Concern for animal welfare is of high importance for the purchase of organic meat and cheese, while concern for healthiness and naturalness is crucial for the purchase of organic frozen food and sweets. However, a challenge for expanding the organic market based on the current core target group of regular organic buyers arises from the fact that these consumers generally have low consumption levels of meat, sweets, and processed foods.

Organic food producers should therefore develop product-category-specific marketing actions. Organic sweets may have a potential for health-concerned consumers, though only if such products do actually have healthier dietary properties than their conventional counterparts, since organic labeling alone seems insufficient to contend with enjoyment as the main motive for buying sweets (McIntyre and Schwanke, 2010). Given that healthy lifestyles typically involve low levels of sweet consumption, the market for organic sweets will probably rather remain a niche market.

This study has confirmed the findings of a previous household panel study by Van Doorn and Verhoef (2015) in showing that the values of quality and enjoyment are not positively related to the purchase of organic food overall. Our results imply that the large segment of quality-conscious consumers is currently not committed to buying organic food. This could partly be due to consumers perceiving the “healthiness” of organic production as reducing the pleasure of consuming these products (McIntyre and Schwanke, 2010; Van Doorn and Verhoef, 2011). We argue that this target group represents an untapped opportunity for organic food producers, since quality-conscious consumers are generally less price-conscious. In order to gain this segment as new organic consumers, organic producers should focus on the high quality of organic products in their marketing communications so as to convince this target group that organic products are of the same or better quality than conventional counterparts.

Another problem with reaching the quality-oriented consumers might be the limited organic product assortment. Our data suggests that consumers who place high importance upon quality and enjoyment tend to prefer conventional cheese. Cheese is a somewhat special product category in that specialty foods play an important role and the selection of organic cheese is often limited. In this case, the solution to reaching the target group of quality-conscious consumers would lie in increasing the organic product assortment to better meet the taste preferences of these consumers. According to Van Doorn and Verhoef (2011), this proposition applies to all hedonic product categories where pleasure and enjoyment plays the decisive role.

### The Mediating Role of Attitudes Within the Value-Behavior Relation

A closer look at the mediating role of attitudes toward organic food within the value-behavior relation in our data provides interesting insights. While we found that food-related values are good predictors of attitudes, certain values also inserted significant direct effects on behavior, suggesting that cognitively formed attitudes do not fully mediate the effect of values on behavior. Interestingly, we found that values related to convenience, quality and enjoyment affected behavior directly (with negative effects on organic purchases), while these values did not influence cognitively formed attitudes toward organic food. This means that even if consumers have developed positive attitudes toward organic products, they tend to behave habitually and perhaps impulsively when it comes to the actual purchase decisions. The desire for convenience and enjoyment is thus a possible explanation for why consumers do not act in accordance with their positive attitudes toward organic food. For example, purchase behavior could be driven by an immediate desire for enjoyment that overwhelms the intention to purchase organic food, especially in the case of certain products like sweets (Hauser et al., 2013) and cheese (Bernabéu et al., 2010).

Values related to local and domestic production were not associated with the purchase of organic food, though they were found to positively influence attitudes toward organic food. This implies that consumers who place value on local food also prefer organic food, though they do not necessarily opt for organic products when it comes to real purchase decisions. One reason for this could be the relatively low availability of local organic products in supermarkets in Germany, while local conventional food is more commonly found in supermarkets.

The findings also show that convenience-oriented consumers do not necessarily have negative attitudes toward organic food. This is an interesting finding in view of the fact that household panel data from 2008 proved a negative relation between convenience orientation and attitudes toward organic food (Janssen, 2018). However, we also found that convenience-oriented consumers buy significantly less organic food than people without convenience orientation, which is also consistent with the findings of Janssen's study (Janssen, 2018). This result suggests that an attitude-behavior gap may exist for some convenience-oriented consumers.

Overall, the present study shows that organic food purchases and attitudes are not completely driven by the same values, and their relative importance differs. Therefore, the results of studies on the effects of food values on attitudes and intentions cannot simply be generalized to real purchase behavior.

### Limitations

To the best of our knowledge, this research study is the first to have analyzed consumer behavior for different organic food categories using household panel data, and therefore, the study extends knowledge on consumer behavior regarding organic food (Van Doorn and Verhoef, 2015; Moser, 2016; Janssen, 2018). Even though the comparison of the models for the different food categories is a valuable contribution to the existing literature, the applied method does not allow checking for statistically significant differences between the models. A further limitation of the research study is that the sample is not directly comparable to the German population according to age and income, and therefore, descriptive data need to be treated with caution. However, since the study's primary aim is to analyze relations between values, attitudes, and behavior, a representative sample is not a decisive aspect in the first place.

Moreover, it was not possible to examine the effects of all influential factors on the complex process of purchase behavior, including factors such as geographical origin, store type, packaging and promotion, and trust in different types of retailers, which might account for parts of the unexplained variance. Moreover, attitudes toward organic food were measured with regard to food consumption in general and not explicitly for the specific food categories. This is important because consumers may have positive attitudes toward organic vegetables and milk but not toward organic sweets or cheese. The attitude-behavior gap within the specific product categories would possibly have been lower if product-specific attitudes had been measured.

The analyzed data covers consumers' actual purchase behavior and therefore offers a high degree of validity as compared to surveys and purchase experiments. However, a recent review study (ElHaffar et al., 2020) on the attitude-behavior-gap in the area of sustainable consumption put forward that more qualitative studies, studies based on experiments as well as consumer segmentation approaches are needed in order to find solutions to close the gap in the future. Moreover, barriers that prohibit the transformation of attitudes into behavior need to be analyzed more deeply in order to extend the market for sustainable products (Yamoah and Acquaye, 2019).

## Data Availability Statement

The datasets presented in this article are not readily available because we got the household panel data from the research institute GfK and are not permitted to share the data.

## Ethics Statement

Ethical review and approval was not required for the study on human participants in accordance with the local legislation and institutional requirements. The patients/participants provided their written informed consent to participate in this study.

## Author Contributions

IS: carried out data preparation, aggregation and anaylsis, and wrote the manuscript. MJ: had the idea for the research project and added substantially to the development of the research question and gave comprehensive feedback to the manuscript and provided knowledge on the method of data analysis, the interpretation of the results, and the discussion and conclusions parts. Both authors contributed to the article and approved the submitted version.

## Conflict of Interest

The authors declare that the research was conducted in the absence of any commercial or financial relationships that could be construed as a potential conflict of interest.
