# Supplementary material for: How and Why Does the Attitude-Behavior Gap Differ Between Product Categories of Sustainable Food? Analysis of Organic Food Purchases Based on Household Panel Data
Source: Front Psychol. 2021 Feb 16;12:595636. doi: 10.3389/fpsyg.2021.595636 (PMC7928379; doi:10.3389/fpsyg.2021.595636)
Supplement: Supplementary file 1 [file Table_1.DOCX]

Supplementary Material.

# Supplementary Tables

Table 1: Direct effects on organic purchases for food overall and different food categories

|  | food overall | | cheese | | meat | | frozen food | | sweets | |
| --- | --- | --- | --- | --- | --- | --- | --- | --- | --- | --- |
| Healthiness and naturalness | 0.03 | * | 0.01 | n.s | -0.01 | n.s. | 0.04 | ** | 0.08 | *** |
| Convenience orientation | -0.10 | *** | -0.07 | *** | -0.04 | ** | -0.09 | *** | -0.03 | * |
| Local and domestic food | -0.07 | *** | -0.04 | ** | -0.02 | n.s. | -0.06 | *** | -0.06 | *** |
| Environmental protection | 0.07 | *** | 0.05 | *** | 0.03 | * | 0.02 | n.s. | 0.07 | *** |
| Quality and enjoyment | -0.05 | *** | -0.04 | ** | -0.01 | n.s. | -0.02 | n.s. | -0.03 | * |
| Price consciousness  (single indicator) | -0.07 | *** | -0.08 | *** | -0.05 | *** | -0.05 | *** | -0.05 | *** |
| Animal welfare  (single indicator) | -0.02 | * | 0.01 | n.s. | 0.11 | *** | -0.05 | *** | -0.06 | *** |
| Attitudes towards organic food | 0.46 | *** | 0.29 | *** | 0.21 | *** | 0.24 | *** | 0.25 | *** |
| Age | -0.04 | *** | -0.01 | n.s. | -0.01 | n.s. | -0.02 | * | -0.05 | *** |
| Education | 0.04 | *** | 0.04 | *** | 0.03 | ** | 0.03 | * | 0.03 | ** |
| Income | 0.05 | *** | 0.04 | *** | 0.02 | n.s. | 0.04 | ** | 0.01 | n.s. |

**** significant at p < 0.001; ** significant at p < 0.01; * significant at p < 0.05, n.s.: not significant*

Table 2: Indirect effects on organic purchases for food overall and different food categories

|  | food overall | | cheese | | meat | | frozen food | | sweets | |
| --- | --- | --- | --- | --- | --- | --- | --- | --- | --- | --- |
| Healthiness and naturalness | 0.15 | *** | 0.10 | *** | 0.07 | *** | 0.08 | *** | 0.08 | *** |
| Convenience orientation | 0.00 | n.s. | 0.00 | n.s. | 0.00 | n.s. | 0.00 | n.s. | 0.00 | n.s. |
| Local and domestic food | 0.08 | *** | 0.05 | *** | 0.03 | *** | 0.04 | *** | 0.04 | *** |
| Environmental protection | 0.08 | *** | 0.05 | *** | 0.03 | *** | 0.04 | *** | 0.04 | *** |
| Quality and enjoyment | 0.01 | n.s. | 0.01 | n.s. | 0.00 | n.s. | 0.00 | n.s. | 0.00 | n.s. |
| Price consciousness  (single indicator) | -0.08 | *** | -0.05 | *** | -0.03 | *** | -0.04 | *** | -0.05 | *** |
| Animal welfare  (single indicator) | 0.18 | *** | 0.12 | *** | 0.09 | *** | 0.10 | *** | 0.10 | *** |
| Attitudes towards organic food |  |  |  |  |  |  |  |  |  |  |
| Age | -0.02 | *** | -0.02 | *** | -0.01 | *** | -0.01 | *** | -0.01 | *** |
| Education | 0.05 | *** | 0.03 | *** | 0.02 | *** | 0.02 | *** | 0.02 | *** |
| Income | 0.02 | *** | 0.01 | *** | 0.01 | *** | 0.01 | *** | 0.01 | *** |
